# Multi-rod posterior correction only with halo-femoral traction for the management of adult neuromuscular scoliosis (> 100°) with severe pelvic obliquity: a minimum 5-year follow-up

**DOI:** 10.1186/s13018-023-04285-4

**Published:** 2023-10-19

**Authors:** Hong-Qi Zhang, Ang Deng, Chao-Feng Guo, Yang Sun, Meng-Jun Li

**Affiliations:** 1 Department of Spine Surgery and Orthopaedics, Xiangya Hospital, Central South University, Xiangya Road 87, Changsha, China; 2 National Clinical Research Center for Geriatric Disorders, Xiangya Hospital, Central South University, Xiangya Road 87, Changsha, China

**Keywords:** Neuromuscular scoliosis, Multi-rod, Pelvic obliquity, Halo-femoral traction, Posterior correction

## Abstract

**Background:**

Many patients with neuromuscular scoliosis (NMS) experience a variety of difficult medical problems that aggravate the development effects of progressive scoliosis and pelvic obliquity (PO). The objective of the current study was to assess the safety and effectiveness of multi-rod posterior correction only (MRPCO) with halo-femoral traction (HFT) for the management of adult NMS (> 100°) with severe PO.

**Methods:**

From 2012 to 2017, 13 adult patients who suffered from NMS (> 100°) with severe PO underwent MRPCO with HFT. The radiography parameters in a sitting position, such as the coronal Cobb angle of the main curve, the PO and the trunk shift (TS), were measured at the preoperative, postoperative and final follow-up stages. The preoperative and final follow-up assessment of the Visual Analogue Scale (VAS) and Oswestry Disability Index (ODI) was taken.

**Results:**

The average follow-up span was 68.15 ± 6.78 months. There was decreased postoperative coronal Cobb angle with an average mean of 125.24° ± 11.78° to 47.55° ± 12.10°, with a correction rate of 62.43%; the PO was reduced to 6.25° ± 1.63° from 36.93° ± 4.25° with a correction rate of 83.07%; the TS was reduced to 2.41 cm ± 1.40 cm from 9.19 cm ± 3.07 cm. There was significant improvement in all parameters compared to the preoperative data. The VAS score reduced from 4.77 ± 0.93 to 0.69 ± 0.75, and the ODI score reduced from 65.38 ± 16.80 to 28.62 ± 12.29 at the final follow-up.

**Conclusions:**

Treatment of adult NMS (> 100°) with severe PO could be safe and effective with MRPCO with HFT. In order to obtain the optimum sitting balance, this could reduce the prevalence of complications and rectify the curvature and the correction of PO.

## Introduction

In patients with neuromuscular disorders, the pervasiveness of severe scoliosis is at the spectrum of 50% to 80% [1–3]. The expansion of the scoliotic curve into the pelvis and the resulting coronal imbalance lead to pelvic obliquity (PO), which required spinopelvic fixation. The severity of these deformities generally depends on both the age of the patient at the onset of the deformity and the depth of the neurological involvement. Moreover, neuromuscular scoliosis (NMS) is distinguished by the aggressive progression of curve during growth and could advance after the skeleton reaches adulthood. The progression of NMS can cause aggravation of sitting imbalance, pain, pressure sores, psychological problems, pulmonary dysfunction and increased mortality [1–6]. Conservative treatment, such as braces and wheelchair modifications, has not been effective for the prevention of curve progression in NMS, which is a challenge for the spinal surgeon [6].

Many surgical options have been described for the management of NMS, but no standard approach has been established [6–11]. Previously, surgical treatment of severe spinal deformities was carried out with different surgical techniques, such as combined anterior release and posterior instrumentation in one or two staged surgeries with different outcomes [7, 8]. Additional anterior approaches, however, had a negative effect on pulmonary function and required longer surgery and anaesthesia times, particularly in patients with NMS. Although vertebral column resection (VCR) could significantly correct the severities of spinal deformities, there were a number of drawbacks, including a significant increase in perioperative complications, blood loss, deterioration of cardiopulmonary function, failure of bone grafting and frequent neurological sequelae [9–11]. Consequently, the objective of this study was to assess the safety and efficacy of multi-rod posterior correction only (MRPCO) with halo-femoral traction (HFT) for the management of adult NMS (> 100°) with severe PO.

## Materials and methods

### Patient data

In this study, we retrospectively evaluate the clinical efficacy of MRPCO with HFT in 13 patients (five males and eight females; age 20–42 years; average age, 29.62 ± 7.12 years) that suffered from NMS (> 100°) with severe PO and were treated in our department between 2012 and 2017. During this period, a total of 42 patients with NMS were treated, and according to the inclusion and exclusion criteria mentioned below, 13 patients were ultimately included in this study. There were six cases of poliomyelitis (Polio), four cases of cerebral palsy (CP) and three cases of spinal cord injury (SCI). All patients had long thoracolumbar or lumbar C-shaped curves, which extend distally to induce severe PO, according to the results of radiography examinations such as X-ray, CT and MRI.

All patients lost their ambulatory ability and were wheelchair-bound. Additionally, they experienced impaired trunk control and balance as a result of acute PO, as well as back pain and trouble sitting, demanding support from the upper limbs. Functional level was described according to the Gross Motor Function Classification System (GMFCS): Level IV in 11 cases and Level V in 2 cases. According to adult NMS, the indications to proceed with surgery are multi-faceted and must involve a shared decision-making approach with the patients and their families: (1) magnitude of the curve more than 50°; (2) deterioration of functional status, such as costo-pelvic or back pain, poor sitting balance, respiratory dysfunction or difficulties with feeding and self-care; (3) progression of the curve; or (4) a combination thereof. The indications for pelvic fixation were PO of > 15° and poor control of the trunk as indicated by a lack of independent sitting or standing. The Xiangya Hospital of Central South University Ethics Committee authorised the study. All proceedings were carried out in accordance with the applicable rules and regulations. For study participation, informed consent was acquired.

The inclusion criteria were as follows: (1) severe NMS with coronal Cobb angle of major curve more than 100°; (2) severe PO more than 30°; (3) age > 18 years; (4) preoperative HFT; (5) posterior-only surgical correction performed using multiple rods; and (6) patients with a minimum follow-up of 5 years who have been treated in our hospital.

The exclusion criteria were as follows: (1) instrumentation without pelvic fixation; (2) non-NMS such as congenital scoliosis, idiopathic scoliosis and degenerative scoliosis; (3) posterior-only correction using two rods; (4) any types of anterior approach before; and (5) inadequate clinical and radiological follow-up documentation.

### Preoperative traction

All patients underwent Xiangya continuous-incremental HFT. The initial traction force applied was 2 kg through distal femur traction to the lower extremity and 2 kg from the halo to the head. If patients tolerated well, 2 kg was increased to the extremity and head daily. Depending on the patient’s tolerance, the maximum traction force applied was from 33 to 50% of the body weight. During traction, neurological function was assessed carefully. The traction was utilised for 18–20 h per day. By the weekly radiographic outcome of curve improvement, the length of the traction period was determined. Traction continued until there was no significant improvement in Cobb angle. Gradual traction was applied for 3–6 weeks. Nutritional supplement treatments were performed during the traction to increase body weight and improve nutritional status. Respiratory training, such as deep respiration and balloon exercise, was applied [12, 13]**.**

### Operative procedure

The somatosensory evoked potential (SEP) and motor evoked potential (MEP) were extensively used to track the spinal cord's activities during the procedure while HFT was maintained. Pedicle screws (or hooks) were placed at the key vertebrae and adjacent to them to provide multiple anchor points after the exposure of posterior spinal components at the designated instrumentation level through a midline incision. Pelvic fixation was done using iliac screws or sacral-alar-iliac (SAI) screws. Intertransverse ligaments, facet joint capsules and constricted soft tissues at the stiff segments were all fully released. One or two short rods across the apical and lumbosacral regions were first placed on the concave side or both sides. Distraction at the concave side and compression at the convex side were adopted for correcting lumbosacral curves and levelling the pelvis as much as possible. Then, two long rods were placed on both sides to correct the remaining coronal and sagittal imbalance and PO. Therefore, multiple rods spread the corrective force and stabilised the spinopelvic construct. The fusion and fixation of all structural curves are essential, and allogenous or autogenous bone grafts could be implanted for fusion [14–16].

Postoperatively, patients underwent a neurological assessment, were mobilised early and started working out while wearing braces 12 days later. On average, all patients wore braces for three months before they were gradually removed.

### Visual Analogue Scale (VAS) and Oswestry Disability Index (ODI)

In preoperative and at the final follow-up, VAS assessed pain without using an analgesic. Additionally, ODI evaluated everyday activities prior to surgery and throughout the final follow-up to analyse clinical performance.

### Radiographical and statistical analysis

The parameters of sitting-position radiography examination, such as the coronal Cobb angle of the main curve, PO, trunk shift (TS) and correction rate, were measured at the preoperative, postoperative and final follow-up stages. The data were analysed with SPSS 22.0 and presented as means ± SD. The parameters preoperatively, postoperatively and at the final follow-up were compared using a paired t-test. A statistically significant difference is shown by *P* < 0.05.

## Results

### Surgical results

The average time of the surgery was 323.85 ± 44.07 min, with a range of 260 to 390 min, and the average blood loss was 1360.77 ± 370.12 ml (range, 910–2230 ml). Each patient underwent a thorough neurological assessment following surgery and at the final follow-up. The mean average follow-up time was 68.15 ± 6.78 months (range, 60–84 months).

No patient died during the operation, and there were no serious side effects such as significant blood vessel damage, spinal cord damage or nerve damage. Additionally, there were no incidences of profound infection, cerebrospinal fluid leakage or newly discovered neurological injury. (Neurological function was consistent with the preoperative stage.) Two cases developed superficial wound dehiscence without infection, managed by a local debridement. One patient experienced pneumonia managed by antibiotics. One case of the prominent iliac screw was not related to local pain or skin breakdown at the final follow-up and was observed without intervention. Instrumentation failure-related issues did not arise. One patient was found to have mild bedsores, which were treated with daily bandages.

During traction, one case developed slight pin loosening of the unilateral femur without intervention. The pin-site superficial infection of the unilateral lower extremity occurred in one patient and was controlled by a dressing change. Three cases experienced locally acceptable pain of the pin site. However, there were no neurological complications with regard to HFT during traction.

### Radiographic results

The preoperative mean coronal Cobb angle of the major curve was 125.24° ± 11.78° (range, 105.8°-149.5°); the mean PO was 36.93° ± 4.25° (range, 30.6°-43.2°); and the mean TS was 9.19 cm ± 3.07 cm (range, 5.3 cm-16.4 cm). After HFT, the average coronal Cobb angle of the main curve was reduced to 75.85° ± 12.65° (range, 56.2°–104.3°) with a mean correction rate of 39.72%. After MRPCO, the postoperative average coronal Cobb angle of the main curve was further decreased to 47.55° ± 12.10° (range, 26.9°–74.6°) with a mean correction rate of 62.43%. The postoperative mean PO was reduced to 6.25° ± 1.63° (range, 4.2°–9.1°) with a mean correction rate of 83.07%. The postoperative average TS, which showed remarkable improvement, was 2.41 cm ± 1.40 cm (range, 0.7 cm-6.2 cm).

**Fig. 1:**
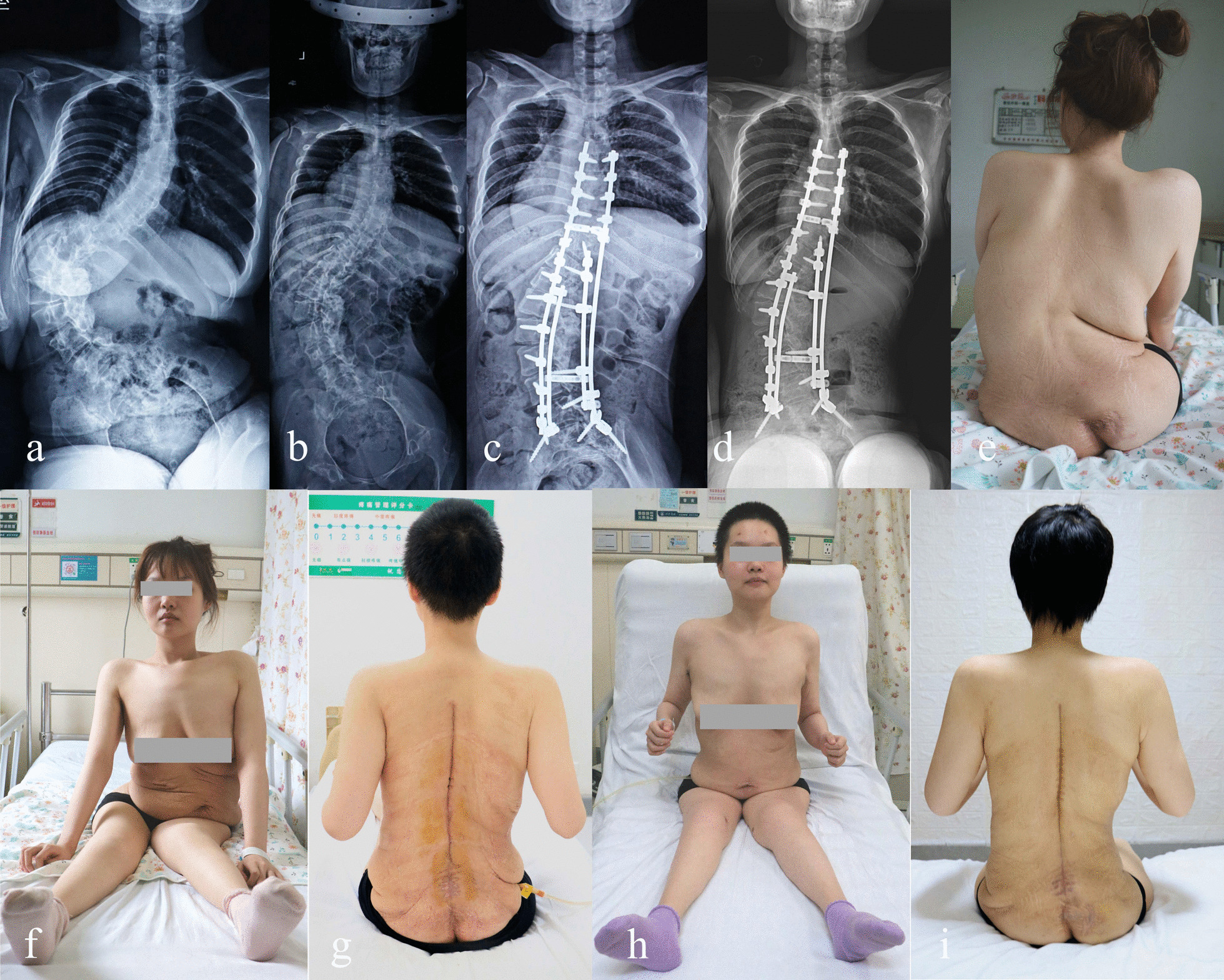
A 25-year-old female with SCI, NMS with severe PO. **a** Preoperative radiographs showed that the coronal Cobb angle of the major curve was 125.4° and PO was 43.2°. **b** After HFT, the coronal Cobb angle was reduced to 87.4° with a correction rate of 30.3%. **c** After MRPCO, postoperative radiographs showed that the coronal Cobb angle was further reduced to 53.8° with a correction rate of 57.1% and PO was reduced to 8.9° with a correction rate of 79.4%. **d** Postoperative radiographs at 66 months after surgery showed that the coronal Cobb angle was 56.4° with a correction rate of 55.02% and PO was 9.4° with a correction rate of 78.24%. **e**–**i** At the preoperative, postoperative and final follow-up stages, the patient’s trunk and sitting balance and cosmetic appearance showed remarkable improvement, which giving greater independence to use her upper limbs for daily activities

The coronal Cobb angle of the major curve and PO was 48.05° ± 12.28° (range, 27.2°–75.1°) and 6.48° ± 1.66° (range, 4.4°–9.4°) at the final follow-up, and the correction loss rates were 0.41% and 0.62%, respectively. The final follow-up mean TS was 2.68 cm ± 1.44 cm (range, 0.9 cm-6.5 cm). There was significant improvement in all the parameters compared to the preoperative data (*P* < 0.001 for all). The patient's sitting and trunk balance improved (Fig. 1; Table 1), giving them more freedom to use their upper limbs for daily tasks.

**Table 1:** Preoperative, postoperative and final follow-up measurement data

Case	Sex	Age (ys)	Diagnosis	Period of follow-up (months)	Duration of surgery (min)	Blood loss (ml)	Pre-OP					After traction		Post-OP					Final follow-up						
							coronal Cobb (°)	PO (°)	TS (cm)	VAS	ODI	Coronal Cobb (°)	Correction rate (%)	Coronal Cobb (°)†	Correction rate (%)	PO (°)†	Correction rate (%)	TS (cm)†	Coronal Cobb (°)‡	Correction rate (%)	PO (°)‡	Correction rate (%)	TS (cm)‡	VAS‡	ODI‡
1	F	25	SCI	66	370	1250	125.4	43.2	6.4	6	74	87.4	30.30	53.8	57.10	8.9	79.40	1.5	56.4	55.02	9.4	78.24	1.9	1	40
2	F	37	Polio	60	380	1960	138.2	41.5	13.1	6	68	83.6	39.51	55.2	60.06	6.4	84.58	2.4	55.7	59.70	6.6	84.10	2.7	1	24
3	M	21	CP	62	320	1540	134.7	40.8	11.6	5	84	76.8	42.98	43.7	67.56	4.5	88.97	3.1	44.1	67.26	4.7	88.48	3.5	1	44
4	M	24	Polio	84	280	1130	115.3	31.9	5.3	3	36	64.3	44.23	41.4	64.09	4.3	86.52	0.7	41.5	64.01	4.4	86.21	0.9	0	10
5	M	33	Polio	68	360	1420	127.9	35.7	7.4	4	52	81.7	36.12	56.6	55.75	6.7	81.23	1.8	56.9	55.51	6.8	80.95	1.9	0	12
6	F	38	SCI	63	340	1170	129.6	39.5	8.7	5	70	73.5	43.29	45.3	65.05	6.5	83.54	2.6	45.6	64.81	6.7	83.04	2.7	1	36
7	F	42	Polio	60	260	910	105.8	30.6	7.8	4	38	56.2	46.88	26.9	74.57	4.2	86.27	0.9	27.2	74.29	4.4	85.62	1.1	0	14
8	F	22	CP	71	290	1080	123.5	37.1	9.4	5	80	69.4	43.81	37.7	69.47	5.3	85.71	2.7	37.9	69.31	5.7	84.64	2.9	1	38
9	M	35	Polio	65	310	1350	118.4	32.8	6.9	4	48	70.6	40.37	48.2	59.29	6.6	79.88	1.6	48.5	59.04	6.8	79.27	1.7	0	14
10	M	20	CP	69	270	1290	116.9	34.4	8.2	4	78	72.1	38.32	36.5	68.78	4.8	86.05	2.4	36.9	68.43	5.1	85.17	2.8	0	32
11	F	27	SCI	70	290	1040	112.3	36.3	7.5	5	72	61.7	45.06	40.8	63.67	6.2	82.92	1.9	41.1	63.40	6.4	82.37	2.3	0	34
12	F	32	Polio	72	390	2230	149.5	33.6	16.4	5	64	104.3	30.23	74.6	50.10	9.1	72.92	6.2	75.1	49.77	9.3	72.32	6.5	2	32
13	F	29	CP	76	350	1320	130.6	42.7	10.8	6	86	84.5	35.30	57.4	56.05	7.7	81.97	3.5	57.8	55.74	7.9	81.50	3.9	2	42

The postoperative and preoperative data as well as the final follow-up and preoperative data were analysed using paired t tests

*SCI* spinal cord injury, Polio poliomyelitis, *CP* cerebral palsy, *PO* pelvic obliquity, *TS* trunk shift, *VAS* Visual Analogue Scale, *ODI* Oswestry Disability Index

*P* < 0.05 implies statistically significant difference. †*P* < 0.05 (postoperative vs. preoperative); ‡*P* < 0.05 (final follow-up vs. preoperative)

### VAS and ODI

VAS and ODI scores were evaluated at the last follow-up and before surgery. The ODI score decreased from 65.38 ± 16.80 (range, 36–86) to 28.62 ± 12.29 (range, 10–44) at the final follow-up, and the VAS score decreased from 4.77 ± 0.93 (range, 3–6) to 0.69 ± 0.75 (range, 0–2). Among all dimensions, personal care, sitting, travelling and lifting scores reduced from 3.85 ± 1.07 to 1.46 ± 1.51, from 3.92 ± 0.76 to 0.23 ± 0.44, from 3.77 ± 1.17 to 0.85 ± 0.80 and from 3.00 ± 1.29 to 1.08 ± 0.86, respectively. All scores revealed significant functional improvements between preoperative and final follow-up scores (*P* < 0.001). There were four cases of CP in this group, and their caregivers fulfilled the outcomes.

## Discussion

Most NMS patients suffer from numerous complicated medical conditions that exacerbate the advancing effects of progressive scoliosis and PO. Some of these issues include back pain, poor trunk balance, sitting intolerance, compromised pulmonary function, hip dysplasia, inadequate nutrition and an increased propensity to decubitus [1–6]. NMS presents a considerable challenge to the spinal surgeon in correcting the deformity and enhancing functional status, particularly in nonambulatory patients. Sitting balance is vital in increasing functional abilities because it gives patients more freedom to use their upper limbs for everyday tasks while enhancing their look, which is important for improving their mental health [5, 6, 17].

Consequently, the main objective of surgery for the NMS is to correct spinal vertebral deformity and prevent the progression of disabling deformity for the reconstruction and restoration of trunk balance. Furthermore, maintain or recreate the sitting balance with a balanced spine on a levelled pelvis by correcting PO and stabilising the lumbosacral junction to prevent nerve lesions and improve the patient’s quality of life [2, 3, 6].

In our study, the mean age of patients was 29.62 years, and the preoperative mean coronal Cobb angle and PO were 125.24° and 36.93°, respectively. The stiffness and rigidity of the curve and difficulties in achieving a satisfactory correction are attributed to the increase in age, coronal Cobb angle and PO. In contrast to the majority of studies, where patients received early correction at around 12 years of age or earlier, at around 70° of Cobb angle and at around 20° of PO, this study's mean age, coronal Cobb angle and PO were much greater [4, 5, 18]. However, the present study achieved acceptable and significant correction in both PO and Cobb angle over a minimum 5-year follow-up period without apparent loss of postoperative correction.

Several procedures have been devised to address severe spinal abnormalities, most notably combined anterior and posterior operation, VCR and preoperative traction followed by posterior correction [7–11, 19, 20]. However, none of the aforementioned methods was regarded as the gold standard for treating severe adult NMS, and they are all still debatable.

The noble method of treating severe and rigid spinal deformities has been regarded as combining anterior and posterior instrumentation techniques [7, 8]. However, both thoracoscopy and open anterior approaches, whether staged or same day, had a worse influence on pulmonary function and potentially contributed to the risk profile and morbidity of perioperative complications, especially in patients with NMS [21, 22]. Moreover, Hero et al. [8] examined the effectiveness of posterior-only instrumentation against a combined method for treating severe scoliosis. Although there was no statistically significant difference between the correction rates of 69% and 66%, the inclusion criteria in their study only took into account curves greater than or equal to 61°, in contrast to our study’s inclusion criteria of severe adult NMS of more than 100°.

Presently, the most effective surgical technique for the correction of severe spinal deformity is VCR because it can achieve a substantial correction rate. In contrast, research by Lenke et al. [10] of 147 patients with severe spinal deformity utilising VCR revealed that these extensive reconstructions were linked to a 59% complication rate, and 39 instances (27%) experienced an intraoperative neurological episode. Other complications include fixation failure due to nonunion, haemopneumothoraxes, haematomas, pulmonary dysfunction and infection [9, 10]. According to the aforementioned factors, VCR could not always be ideal for patients with severe adult NMS compounds with complex medical issues.

Preoperative traction has been used routinely as an adjunctive treatment for severe spinal abnormalities before final surgical repair. It allows the primary and compensation curves to correct partially, with less force and safely, so that definitive surgical correction can occur in a less severe curve. With fewer neurological and cardiopulmonary consequences related to definitive surgical repair, these effects can make it easier and better for well-defined correction of spinal deformity [19].

The current traction methods include halo-pelvic traction (HPT), halo-gravity traction (HGT) and HFT. HPT could be continuous without interruption, but it underwent great trauma in the fixation of the pins and was inconvenient for daily nursing and sleep [19]. HGT was the frequently applied traction by using body weight for counterforce, and it could be used when patients were in a wheelchair or bed. However, the outcomes of HGT were apparently different and often controversial on curve correction. Koller et al. [23] found that preoperative HGT could not lead to meaningful release efficacy, and there was no significant difference between the flexibility during HGT and the flexibility on Cotrel traction or bending radiographs. Without prior posterior and/or anterior release, HGT could not be expected to improve severe spinal deformities. Sponseller et al. [24] reported no statistically significant difference between the treatment of severe spinal deformities with and without HGT in major curve correction rate, blood loss, complication rate and operative time. These findings indicated that the actual efficacy of HGT may be overestimated in severe and rigid spinal deformities.

Moreover, HFT was popular and often used. According to Hamzaoglu et al. [20], the average improvement on the major sagittal curve was 53%, the major curve was 51% and the compensatory curve was 33%. HFT improves pulmonary function by lengthening both the thoracic cavity and the spine. The hefty HFT used before surgery may offer stronger traction forces, greatly increase curve flexibility and enhance spinal compliance, allowing a simpler and better overall correction and preventing the screw from pulling out. Notably, in NMS with neurological dysfunction, initial slow skeletal traction prior to surgery may increase the spinal cord's tolerance to stretch trees and ischaemia following curvature rectification. HFT is a foundation for intraoperative orthopaedic treatments and reduces the risks of neurological problems; the patient's neurological status is often examined and evaluated. Compared to HGT, HFT could offer more efficient correction of severe spinal deformities, and these factors were considered as follows [25, 26]: (1) HFT provided more traction forces and better release for both major and compensatory curves and required less traction time compared to HGT; (2) HFT could let patients obtain more effective traction time and efficacy, especially under the condition of sleep or muscle relaxation; and (3) HFT could be maintained during surgery for further release and correction,

Therefore, to avoid the risks of undergoing anaesthesia and surgery more than once as well as the drawbacks of VCR and combined procedure, such as increased operation time, blood loss, frequent neurological complications, aggravation of pulmonary dysfunction and high level of technical requirements, MRPCO with HFT was performed.

The postoperative coronal Cobb angle of the main curve was decreased to 47.55° with a correction rate of 62.43% in this study with severe adult NMS of more than 100°. With a correction rate of 83.07%, the postoperative PO was decreased to 6.25°. These correction rates were comparable to Lenke's VCR [10] and greater than the VCR reported by several publications [2, 9, 27]. However, compared to what was described in the literature [7, 9, 10, 27], blood loss, incidence of complications, difficulty of the procedure, operation time and neurological events were all much reduced.

Traditionally, curves with a Cobb angle of more than 100° are stiffer than smaller deformities, where a two-rod build can frequently produce an acceptable outcome. Previously, two long rods were also usually used to achieve the goals of both global balance maintenance and deformity correction in adult spinal deformity (ASD) patients. Howbeit, the translation of the rods during installation may potentially influence their biomechanical stability even after osteotomy, and the rod installation was still difficult. Furthermore, the global coronal balance might not be preserved, and the biomechanical stress would rise if the rods were forced too hard to fit the screws. Although, with only two long rods built, it was also challenging to control both coronal and sagittal global balance, which could result in global imbalance and possibly implant failure [1, 28].

The MRPCO approach involved breaking down the correction processes for severe, complicated deformities into separate steps. Each step included one or two manoeuvres and was solely concerned with a specific task. The MRPCO could reduce rod installation difficulty by separating manoeuvres and multi-rod systems. The spine could be stabilised gradually, and the multi-rod build's final biomechanical properties were better than the conventional two-rod design [29]. As a result, the MRPCO could divide the intricate adjustment into a number of straightforward surgical techniques that were simpler to carry out.

Moreover, the use of a short lumbosacral and apical concave satellite rod prevented the long concave rod from bending excessively during MRPCO, which in turn reduced ligamentous buckling, permitted precise management of the correction and may eventually lower the risk of pseudoarthrosis [1, 30]. The multi-rod construction also established a progressive transition zone from the stress concentration region to the uninstrumented region, distributing the corrective force of each rod at the apical and/or lumbosacral region.

According to Merrill et al. [31], lumbosacral pseudoarthrosis with implant failure occurred statistically more frequently in ASD patients with conventional two-rod constructs to the pelvis than in patients with multi-rod constructs. This implied that multiple rods may address mechanical instability, not biology, as the primary cause of failure. The multi-rod structures could significantly lessen the stress on the spinal fixators at the site of the lumbosacral osteotomy, according to finite element models [32]. Furthermore, the stiff construct-based gradual MRPCO of the spine has the benefit of avoiding some of the dangers associated with other alternative procedures while also having the ability to undo each incremental correction in response to changes in MEP. In this study with severe adult NMS of more than 100°, the correction rate (62.43%) of MRPCO with HFT was still obviously higher than that (range, 46.69%-53.63%) of multi-rod constructs reported by some authors [1, 28].

In a previous article on NMS, O' Brien et al. [17] recommended that levelling the pelvis should be the patient's first priority when scoliosis and PO coexist due to the patient's fundamental functional needs of sitting and walking. Due to these factors, treating PO in these patients is just as crucial as treating spinal deformities. Miladi et al. [33] also noted that, regardless of the residual Cobb angle following surgery, one of the essential goals of NMS correction and pelvic fixation was the reconstruction of a three-dimensional global trunk balance in an ideal position for fusion.

Moreover, we considered the following benefits of MRPCO with segmental spinopelvic pedicle screw fixation: (1) achieve strong pelvic stability and further adjust the PO after the cantilever manoeuvre; (2) there was no need to postoperatively immobilise the patient because of the extremely stiff multi-rod construct; and (3) because the segmental curves were corrected with multiple rods, the two long rods were only in charge of controlling overall balance over a levelled pelvis. In this study, in comparison with the previous published series, our average PO and TS were more significant than most maximum POs and TSs in other investigations. Our correction rates of 83.07% and 73.78%, respectively, were comparable and, in some cases, better than those reported in other series published despite the added severity [2, 4, 18, 34, 35]. This was partly ascribed to MRPCO with HFT and improved pelvic control with segmental spinopelvic fixation.

Some limitations need to be taken into account. The included cases had a small sample size. The outcome of the longer follow-up and relatively homogeneous population should be investigated further. Future prospective comparative studies may provide further insight into these procedures’ advantages and potential fallacies.

## Conclusions

In this study, MRPCO with HFT could be effective and safe for the management of adult NMS (> 100°) with severe PO. It could limit the prevalence of complications further, improve the curve and PO correction rate and achieve ideal sitting balance, giving patients more freedom to use their upper limbs for daily tasks.

## Data Availability

The data sets used and/or analysed during the current study are available from the corresponding author on reasonable request.

## Abbreviation

PO: Pelvic obliquity
NMS: Neuromuscular scoliosis
VCR: Vertebral column resection
SCI: Spinal cord injury
CP: Cerebral palsy
Polio: Poliomyelitis
MRPCO: Multi-rod posterior correction only
HFT: Halo-femoral traction
VAS: Visual Analogue Scale
ODI: Oswestry Disability Index
CT: Computed tomography
MRI: Magnetic resonance imaging
TS: Trunk shift
MEP: Motor evoked potential
SEP: Somatosensory evoked potential
SAI: Sacral-alar-iliac
ASD: Adult spinal deformity
GMFCS: Gross Motor Function Classification System
